# A Digital Health Innovation to Prevent Relapse and Support Recovery in Youth Receiving Specialized Services for First-Episode Psychosis: Protocol for a Pilot Pre-Post, Mixed Methods Study of Horyzons-Canada (Phase 2)

**DOI:** 10.2196/28141

**Published:** 2021-12-07

**Authors:** Shalini Lal, John F Gleeson, Simon D'Alfonso, Geraldine Etienne, Ridha Joober, Martin Lepage, Hajin Lee, Mario Alvarez-Jimenez

**Affiliations:** 1 School of Rehabilitation Faculty of Medicine University of Montréal Montréal, QC Canada; 2 Youth Mental Health and Technology Lab Health Innovation and Evaluation Hub University of Montréal Hospital Research Centre Montréal, QC Canada; 3 Prevention and Early Intervention Program for Psychosis Douglas Mental Health University Institute Montréal, QC Canada; 4 ACCESS Open Minds Douglas Mental Health University Institute Montréal, QC Canada; 5 Healthy Brain and Mind Research Centre and School of Behavioural and Health Sciences Australian Catholic University Fitzroy Australia; 6 School of Computing and Information Systems University of Melbourne Parkville Australia; 7 Department of Psychiatry McGill University Montréal, QC Canada; 8 Centre for Youth Mental Health University of Melbourne Parkville Australia; 9 Orygen Parkville Australia

**Keywords:** psychotic disorders, mental health, telemedicine, young adult, mental health services, e–mental health, virtual care, schizophrenia, eHealth, social support, therapy, psychiatry, psychology

## Abstract

**Background:**

Psychotic disorders are among the most disabling of all mental disorders. The first-episode psychosis (FEP) often occurs during adolescence or young adulthood. Young people experiencing FEP often face multiple barriers in accessing a comprehensive range of psychosocial services, which have predominantly been delivered in person. New models of service delivery that are accessible, sustainable, and engaging are needed to support recovery in youth diagnosed with FEP.

**Objective:**

In this paper, we describe a protocol to implement and evaluate the acceptability, safety, and potential efficacy of an online psychosocial therapeutic intervention designed to sustain recovery and prevent relapses in young adults diagnosed with FEP. This intervention was originally developed and tested in Australia and has been adapted for implementation and evaluation in Canada and is called Horyzons-Canada (HoryzonsCa).

**Methods:**

This cohort study is implemented in a single-center and applies a pre-post mixed methods (qualitative-quantitative convergent) design. The study involves recruiting 20 participants from a specialized early intervention program for psychosis located in Montreal, Canada and providing them with access to the HoryzonsCa intervention for 8 weeks. Data collection includes interview-based psychometric measures, self-reports, focus groups, and interviews.

**Results:**

This study received funding from the Brain and Behavior Research Foundation (United States), the Quebec Health Research Funding Agency (Canada), and the Canada Research Chairs Program. The study was approved by the Research Ethics Board of the Centre intégré universitaire de santé et de services sociaux de l'Ouest-de-l'Île-de-Montréal on April 11, 2018 (#IUSMD 17-54). Data were collected from August 16, 2018, to April 29, 2019, and a final sample of 20 individuals participated in the baseline and follow-up interviews, among which 9 participated in the focus groups. Data analysis and reporting are in process. The results of the study will be submitted for publication in 2021.

**Conclusions:**

This study will provide preliminary evidence on the acceptability, safety, and potential efficacy of using a digital health innovation adapted for the Canadian context to deliver specialized mental health services to youth diagnosed with FEP.

**Trial Registration:**

ISRCTN Registry ISRCTN43182105; https://www.isrctn.com/ISRCTN43182105

**International Registered Report Identifier (IRRID):**

RR1-10.2196/28141

## Introduction

### Background

Psychotic disorders have a lifetime prevalence of 3% [1] and include symptoms such as delusions, hallucinations, disorganized thoughts and behaviors, poverty of thought and affect, apathy, and deficits in verbal memory and executive functioning [2,3]. The onset of psychosis typically occurs during adolescence or young adulthood (ie, between the ages of 15-25 years), often leading to substantial impairments in social and community functioning, and ultimately derailing transitions toward life goals. Psychosis is associated with three of the top five leading causes of disability in the world (ie, major depression, bipolar disorder, and schizophrenia) for adolescents and young adults [4], incurring substantial costs for the health care system and society in terms of loss of productivity. As such, psychotic disorders have been described in the literature as among the most scientifically challenging and disabling mental disorders [5,6].

Over the past two decades, a specialized early intervention approach has been developed for youth diagnosed with first-episode psychosis (FEP) with the ultimate goal of achieving symptom remission, relapse prevention, and social recovery [7,8]. Many of these programs accept patients with schizophrenia spectrum psychoses and affective psychoses. Specialized early intervention services typically involve medication, psychosocial services (eg, illness education, lifestyle management, employment and education support, or family education and support), and case management [7,8]. The short-term (ie, 1-2 years) benefits of this specialized approach compared to routine care have been reported in several randomized controlled trials, quasi-experimental studies, and reviews of the literature [9-11]. Randomized controlled trials have confirmed that young people treated in a specialized early intervention service have higher rates of adherence; lower relapse and hospitalization rates; better quality of life; and improved outcomes at 12, 18, and 24 months compared to patients in routine care [11-13].

Although the specialized early intervention approach has shown promise in improving outcomes in youth with FEP, the need to develop better psychological, social, and vocational interventions for this population continues to be a priority. This is because there are several clinical challenges associated with treating this population that are of high concern for clinicians, patients, and families. These include high rates of relapse (estimated at 30%) [14,15] and high rates of service disengagement, which range between 20% to 40% across specialized early intervention programs [16,17]. There are also challenges in sustaining improvements in symptoms and global functioning beyond the first 2 years of receiving specialized services [18].

To date, specialized early intervention services have been restricted to models of care that are predominantly delivered in person. However, research suggests that after an initial period of receiving intensive treatment, some youth begin to experience the phenomenon of *overengagement* or *engulfment* by services and prefer a less intensive form of follow-up [17,19]. Moreover, relying entirely on services delivered in person may not be feasible given that specialized early intervention is resource-intensive, with typical case manager-to-patient ratios being 1:25 and services often delivered in the community, which presents a range of challenges including, for example, loss of productivity and lack of access to transportation [20]. Research is needed on new models of service delivery that are accessible, sustainable, engaging, and effective in supporting recovery in youth diagnosed with FEP. This is especially relevant during the first 5 years of the illness as this phase is considered as the “critical period” when clinical and psychosocial interventions have the highest impact [21]. There is also a need for sustainable models of service delivery that can meet the need for psychosocial support beyond the time frame of receiving specialized early intervention for psychosis services.

Information and communication technologies (ICTs) offer a promising avenue for addressing the aforementioned clinical challenges by increasing access and quality of mental health services, and ultimately supporting the process of recovery for youth experiencing FEP [22-25]. These technologies can potentially deliver services using a less intensive and more engaging format that is compatible with the culture of young people growing up in the 21st century. Our prior research conducted in a Canadian specialized early intervention for psychosis setting has shown that more than 90% of youth diagnosed with FEP receiving specialized services have regular access to the internet through computers and mobile devices, and many are receptive to the idea of using these technologies for mental health care [24,25]. However, despite the potential of technology for improving mental health care for youth, this area has received limited research attention in the Canadian field of early intervention for psychosis. Consequently, few studies exist on the acceptability, safety, and efficacy of digital health innovations to support recovery in youth diagnosed with FEP. Country-specific research on digital mental health care is relevant for advancing policy and practice that is contextualized to the needs of a nation’s population. For example, the Canadian health care landscape has particular characteristics that justify locally driven digital mental health service research. According to Martin and colleagues [26], Canada has a health care system that is managed through provincial and territorial insurance plans; a vast geography; and a significant population diversity in terms of density, culture, and language [26]. These characteristics are important considerations for adapting, implementing, and evaluating digital health service innovations in the Canadian context.

### The Horyzons Intervention Platform

Members of our team have developed an innovative web-based therapeutic intervention platform, called Horyzons, designed to sustain the treatment benefits of early intervention for psychosis and to promote long-term social functioning [27]. This intervention delivers evidence- and strengths-based targeted psychosocial interventions and is enhanced by a moderated online social networking environment. Youth diagnosed with FEP are guided through interactive games to identify, discuss, and develop key personal strengths in an online environment and in real life to address relapse risk factors and psychological well-being.

Specifically, Horyzons consists of *interactive psychosocial interventions* that are informed by evidence-based psychosocial interventions targeting key risk factors and salient domains in the early recovery process (including psychoeducation, vocational recovery, early warning signs of relapse, depression, social anxiety, personal strengths). Youth with FEP are prompted to practice newly acquired skills through over 350 purpose-developed behavioral activities, which are designed to bridge the gap between online therapy and real-world outcomes (eg, tips on how to use personal strengths to cope with stress, enhance well-being, or improve social connectedness). Horyzons also includes *peer-to-peer web-based social networking* that includes a web feed (or news feed) where youth with FEP and moderators can post comments and information, upload pictures and videos, and *like* different types of content, and includes *moderation* that is conducted by clinicians and peer support workers.

Clinician moderators provide guidance, monitor clinical status, and ensure safety of the social networking environment. Clinician moderators develop brief case formulations that are presented at weekly supervision meetings with the senior clinical research team. Clinician moderators send each youth tailored content suggestions (eg, step or action) weekly based on the young person’s needs, interests, and strengths. Suggestions appear on the young person’s home page, and they receive a text notification via the intervention platform’s SMS text messaging feature. Peer support moderators are young adults with lived experience who have received peer support training and who have been stable and in remission for a minimum of 2 years. Their role includes assisting with orientation to the Horyzons intervention platform, providing support, and fostering engagement. For example, peer support moderators reach out to new users or users that have not logged into the intervention platform after a week. They also post *icebreakers* and comments, *like* the posts of users, and engage in role modeling activities. They receive supervision from the clinical research team. The integrity of the moderation is ensured through a detailed moderation manual, and all moderators participate in weekly to biweekly group supervision sessions with senior members of the team. The intervention platform includes a comprehensive safety protocol following best practices in internet research involving vulnerable people [28], which considers 3 levels of security (ie, online safety, clinical safety, and system security). This safety protocol (including criteria and the process for withdrawal from the study) was successfully used in previous research on Horyzons [27,29].

### Research and Development of Horyzons and Canadian Adaptations

A detailed description of the intervention, its development, and its subsequent adaptation for the Canadian context can be found in our previous published work [30-32]. The first version of Horyzons was developed iteratively over a 30-month period following participatory design principles involving patients, clinical psychologists, computer programmers, health informatics experts, web and graphic designers, and professional writers. It was then pilot-tested on a sample of 20 young adults for its feasibility, acceptability, utility, and safety considerations over 1 month [27]. Results showed that there were no dropouts and incidents (ie, adverse events or inappropriate use) during the pilot, and system use was high, with a total of 275 log-ins. Specifically, 70% of participants used the system for at least 3 weeks, 95% used the social networking features, and 60% completed at least 3 therapy modules. Moreover, all participants found the system easy-to-use, 70% considered it to be a useful long-term treatment option beyond discharge from early interventions services, 100% considered it to be safe, 85% would recommend it to others, and 100% reported moderation to be helpful. With respect to potential clinical improvements, 70% considered the therapy modules to be helpful, and 60% reported that Horyzons significantly increased their social connectedness. Paired samples *t* tests also revealed a significant reduction in depressive symptoms at follow-up (d=0.60; *P*=.02) [27]. The safety strategy pertaining to the intervention study addressed both static (eg, personality trait variables) and dynamic factors (mental health status), and results indicated no clinical or security problems related to the use of Horyzons during the pilot study [27,33]. Additionally, a second version of Horyzons was evaluated in Australia within the context of a 5-year follow-up randomized controlled trial [29], with results recently published. Horyzons has also been piloted in the United States, and preliminary analyses of within-group effect sizes demonstrated the greatest improvements in psychotic symptoms (d=0.65-0.81), followed by depressive symptoms (d=0.14-0.30) and social functioning (d=0.18) [34]. Horyzons has also been adapted for young people at ultra-high risk for psychosis, and preliminary research suggests that it is effective in improving social functioning in this population [35].

We recently completed a phase 1 adaptation study of the Horyzons intervention platform in two specialized early intervention clinics for FEP in 1 urban and 1 urban-rural setting, in 2 Canadian provinces [30]. The phase 1 study involved a mixed-methods approach, combining descriptive qualitative and quantitative methods, and was informed by the literature on adaptation of psychosocial interventions [31,36]. Data was collected using focus groups, semistructured interviews, and consultations. The main purpose of the phase 1 study was to determine the types of adaptations needed to the website and the therapeutic intervention protocol before implementing the intervention live. Considering participants’ and clinicians’ perspectives on the platform, we adapted the Horyzons intervention for Canada (HoryzonsCa). Specifically, adaptations were made to content on employment, study, and volunteer opportunities, and postdischarge information (eg, finding a family doctor); general use and safety features (eg, terms of use, translation of Australian list of problem words to French, safety protocol); and clinical moderation protocol (eg, clinical notes and supervision structure). However, in this phase 1 study, participants had limited access to all the components of the HoryzonsCa intervention; for example, they had access to the therapeutic content modules but not to clinical and peer support moderation nor were they able to communicate with other participants. Their access was also time-limited to 2 weeks. Thus, we designed this phase 2 pilot protocol to assess the acceptability, safety, and potential efficacy of the HoryzonsCa intervention with all features of the intervention accessible to a target sample of 20 to 25 participants over 8 weeks.

In this paper, we describe the research protocol informing this phase 2 live pilot study of HoryzonsCa. The protocol is largely informed by the Technology Acceptance Model (TAM) [37,38], which is a well-established model focusing on individual perceptions pertaining to ease of use and usefulness of a technology, and the eHealth Adaptation Framework [31], which considers broader factors such as language, culture, and context pertaining to localized implementation of an eHealth innovation. Our attention to the adaptation aspects of Horyzons is in alignment with strategies recommended in the literature to better capture complexities pertaining to the implementation of eHealth innovations [39]. Moreover, during our analysis and interpretation of the results, we will also begin to consider models stemming from sociotechnically informed theories of change at the levels of individual, organization, and system, such as the nonadoption, abandonment, spread, scale-up, sustainability framework, which is intended to facilitate reflection on factors influencing adoption, nonadoption, abandonment, spread, scale-up, and sustainability of eHealth technologies [40]. Such reflections will be important to inform the design of a scaled-up evaluation of the intervention, including the types of data that will be important to collect.

### Objectives and Hypotheses

The aim of this study is to determine the acceptability, safety, and potential efficacy of HoryzonsCa in supporting recovery in young adults receiving specialized services for FEP. Specifically, our objectives are:

- To determine the acceptability of an online therapeutic platform (HoryzonsCa). *Primary hypothesis:* HoryzonsCa will be acceptable to patients. Our conceptualization of acceptability is partially informed by the TAM [37,38], which posits that attitudes toward using a technology influence intention (“acceptance”) to use a technology, which is a direct determinant of behavior [37,38]. The attitude construct is composed of perceived usefulness (an individual’s perception that using an information technology [IT] system will be helpful to them) and perceived ease of use (an individual’s perception that using an IT system will be free of effort) [38]. Patients’ acceptance regarding the use of technologies in health care delivery has been extensively assessed using the TAM model and its derivatives, which highlight the importance of considering factors beyond the individual’s attitudes [41-44]. As such, we also include the concept of adoption in our conceptualization of acceptability, which “is defined as the intention, initial decision, or action to try or employ an innovation or evidence-based practice” [45]. Thus, using this combined understanding of acceptability, in this study, the intervention will be considered acceptable if at least 70% of participants provide positive reports on general experience of the platform, 60% provide positive reports on perceived usefulness (helpfulness), 60% provide positive reports on ease of use, and 60% log onto the site at least 4 times over the 8-week follow-up. These percentages are hypothesized based on the results obtained from the original Horyzons pilot study [27]. We will also determine acceptability through qualitatively assessing perceptions of HoryzonsCa in relation to likes, dislikes, perceived barriers and facilitators to using the platform, and ease of use/experiences of navigating and using its key features (eg, various psychoeducational modules or café) and through exploratory analysis of website use analytics.
- To assess the safety of the HoryzonsCa platform. *Secondary hypothesis:* HoryzonsCa will be safe, defined as no adverse events, reports, or incidents (eg, hospitalization, suicidal ideation, or disclosure to treatment team regarding harm) related to use of the platform from baseline assessment to 8 weeks follow-up, and at least 70% of participants report that they agree or strongly agree with the perceived safety of the platform and perceived confidentiality of information shared on the platform. We will also determine safety through qualitatively assessing perceptions of the HoryzonsCa platform (eg, experiences, concerns).
- To assess the potential efficacy of the HoryzonsCa intervention/platform. *Secondary hypotheses:* Participants will show moderate to large improvements (Cohen *d*≥0.5) on measures of social functioning (Social and Occupational Functioning Assessment Scale; Personal and Social Performance Scale) and either improvement of 1 point or no deterioration on the Clinical Global Impression Scale, from baseline to 8 weeks follow-up, as our primary foci for assessing potential efficacy. We will also assess for improvements in social support, self-esteem and perceived strengths, and symptoms. The analysis will help determine pre-post effect sizes on a number of variables conceptually targeted by the platform. These effect sizes will be used in a future clinical trial (eg, randomized controlled trial) for the purpose of statistical power calculation and estimation of sample size.

## Methods

### Study Design and Setting

This cohort study is implemented in a single center and applies a pre-post mixed methods (qualitative-quantitative convergent) design. Participants are recruited from Prevention and Early Intervention Program for Psychosis (PEPP)–Montreal, the Douglas Mental Health University Institute in Montreal, Quebec. This program provides a comprehensive range of services for young people diagnosed with FEP and follows best practice guidelines for real-world settings [7,8]. Treatment includes psychiatric evaluation and follow-up, modified assertive case management tailored to meet the needs of young patients in the early phase of illness [8] including support toward treatment goals (eg, illness education, return to work or school, and crisis intervention), family support and intervention, and psychosocial group interventions (eg, physical, recreational, recovery, and support).

### Participants, Sample Size Considerations, Recruitment, and Orientation Process

The target sample is 20 to 25 participants that are patients receiving services from PEPP-Montreal. This sample size was determined by the following factors: feasibility of participant recruitment within a 2- to 3-month time frame, budget related to staffing of moderators, and ensuring adequate number of active users on the platform for the social networking features to function effectively. This sample size has also shown to be sufficient to pilot-test the feasibility and acceptability of Horyzons, as illustrated in the prior pilot study [27] while also acknowledging the limitation of this small sample size for efficacy testing (which is not the main objective of this pilot study). Participant inclusion and exclusion criteria are presented in Textboxes 1 and 2, respectively.

**Inclusion criteria.**

**Participants**

- Diagnosis of a psychotic disorder (including affective or nonaffective psychoses) by a clinician
- Receiving specialized services for a first-episode psychosis at the recruitment site
- Considered symptomatically stable and capable of interacting on the online platform and participating in focus groups and semistructured interviews, as judged by their primary clinicians (ie, psychiatrist, case manager)
- 18 years or older
- At low or at most moderate severity score (4 or below) on the suicidality item of the Brief Psychiatric Rating Scale, version 4 [46] for the month preceding study entry. This criterion is to minimize risk for the need of urgent intervention regarding comments pertaining to suicidal ideation and plans posted on the social network or to the moderation team (given that the platform is not monitored 24/7) and to reduce potential anxiety or distress in other participants from overexposure to content focused on suicide.
- Able to nominate an emergency contact

**Exclusion criteria.**

**Participants**

- Intellectual disability
- Hospitalized at the time of recruitment
- Unable to speak or read English
- Diagnosis of antisocial personality disorder or borderline personality disorder
- In the acute phase of mania or psychosis to the extent that their mental status may soon require hospitalization or would impede the participant’s ability to provide informed consent or to participate in interviews and focus groups

In terms of the recruitment process, a member of the research team met with clinicians at the recruitment site to describe the project, including the rationale, objectives, inclusion and exclusion criteria, and methods. The treating clinicians screened all participants. The treating clinicians referred patients who fulfilled the aforementioned inclusion and exclusion criteria. The treating clinicians provided a copy of the study information handout to prospective participants when introducing the study so that they had time to consider the study and ask questions about it. Once participants completed screening for eligibility, were identified as being eligible to participate, and expressed interest to learn more about the study, they were contacted by a member of the research team to receive further details about the project, their participation, and the informed consent process. A study information brochure was provided to participants as a summary of the information contained in the consent form for user-friendly access to key information about the project.

Participants received detailed information concerning their participation including date, time, and duration of scheduled meetings, interviews, and activities related to study participation. In addition, they were contacted 24 hours prior to any scheduled interviews to confirm their participation. They were contacted according to their preferences either by phone, e-mail, or in person when attending the clinic. After providing written informed consent, the research assistant administered the baseline measures, the results of which were also used to confirm clinical stability and eligibility to participate. In a subsequent HoryzonsCa Orientation Meeting, participants were oriented to the HoryzonsCa website and provided with login information. Participants were then able to access the website, at their convenience, over a period of 8 weeks. They were encouraged to log into the website at least 1 time per week for a minimum of 15 minutes per visit. Access to HoryzonsCa was in addition to the services that participants already receive from PEPP-Montreal. The Horyzons safety protocol [27] was adapted for this study and included an assessment procedure for risk of suicide attempt. This assessment has been implemented by members of the team in the context of other psychosocial intervention research projects with the same clinical population. All research team members were trained in the safety protocol. Given that it was the first time the website and safety protocol was used in a Canadian context, the results of this study will help identify potential modifications to improve the safety protocol for future research on HoryzonsCa. The details of the adapted safety protocol and withdrawal criteria are provided in Multimedia Appendix 1.

### Data Collection

We collected quantitative and qualitative data through interview-based psychometric measures, self-reports, focus groups, and interviews. An initial interview consisted of completing a self-reported sociodemographic questionnaire, the Technology Access, Use, and Competency Questionnaire (TAUC-Q), and a combination of self-reported and interviewer-administered clinical measures (for social functioning, global improvement and therapeutic response, social support, self-esteem and perceived strengths, and symptoms). The TAUC-Q and the clinical measures were also administered during the exit interview at the 8-week follow-up along with the interviewer-administered Horyzons-Canada Acceptability, Usability, Safety, and Impact Questionnaire (HC-AUSI-Q). Participants were also invited to a HoryzonsCa Meet-up and Focus Group during the follow-up period. Multimedia Appendix 2 provides an overview of the schedule of assessments that took place across the duration of the follow-up.

### Outcome Measures

#### Sociodemographic Characteristics and Access and Use of Technology

Participants were asked to complete a sociodemographic questionnaire (self-report) consisting of nine questions regarding gender, age, length of service use, highest level of education completed, ethnicity, vocational status, living situation, marital status, and annual income. Participants’ access, use, and attitudes in relation to technology was assessed using the TAUC-Q (self-report), which includes 10 questions regarding participants’ access and use of internet and mobile technology (eg, smartphone, computer, social media, text, or email) and perceived competency of technology. Their responses on these questionnaires will allow us to estimate the transferability of our results and to better understand participant experiences, use, and perceptions of the platform.

#### Acceptability

*Acceptability* (TAM components of perceived ease of use and perceived usefulness) was measured through the HC-AUSI-Q and website use analytics. The HC-AUSI-Q is an adaptation of questionnaires used in the HoryzonsCa phase 1 adaptation study [30,31], the Horyzons Usability Questionnaire from the original Horyzons pilot study [27], and the Website Analysis and Measurement Inventory [47]. The interviewer-administered HC-AUSI-Q consists of a questionnaire and a semistructured interview that includes 16 close-ended and 10 open-ended questions on perceived ease of use, perceived usefulness, enjoyment, and safety. The questions pertaining to acceptability address the topics of general experience (eg, “I had a positive experience on Horyzons-Canada”), usefulness (eg, “Horyzons was useful to identify my warning signs for relapse”), and ease of use (eg, “Overall, the platform is easy to use”). Acceptability in terms of adoption was assessed using website use analytics (eg, frequency of log-ins and patterns of use over 8 weeks).

#### Safety

*Safety*, which considers 3 levels of security (ie, online safety, clinical safety, system security), was assessed using two specific questions (ie, “I felt safe on Horyzons-Canada” and “I felt like the information shared on Horyzons-Canada was confidential”) in the HC-AUSI-Q. In addition, any adverse events, reports, or incidents (eg, hospitalization, suicidal ideation, or disclosure to treatment team regarding harm) in relation to the use of the online system were carefully monitored and quantified over the study duration. The causal relationship between adverse events and use of HoryzonsCa was determined based on detailed documentation of each event and through discussion by members of the research and moderation team. All adverse events, reports, and incidents (eg, hospitalization, major deterioration in ability to function) were also submitted to the ethics review board for further examination.

#### Potential Efficacy

In terms of *primary clinical measures*, *social functioning* was measured using the interviewer-administered Social and Occupational Functioning Assessment Scale that comprises a 100-point single item to assess social and occupational functioning [3] and the interviewer-administered Personal and Social Performance Scale that consists of a 100-point single item to assess functioning in four domains: socially useful activities (including work and study), personal and social relationships, self-care, and disturbing and aggressive behaviors [48]. In terms of *secondary clinical measures*, *global improvement and therapeutic response* was assessed using the clinician-administered Clinical Global Impression Scale that consists of two items to assess global improvement and severity of illness [49]. We also assessed *social support* using the self-reported Multidimensional Scale of Perceived Social Support that includes 12 items (eg, “I get the emotional help and support I need from my family) [50]; *self-esteem and perceived strengths* using the self-reported Self-Esteem Rating Scale that consists of 40 items (eg, “I feel that I am a very competent person”) [51], the self-reported Strengths Knowledge Scale that includes 8 items (eg, “I know what I do best”) [52], and the self-reported Strengths Use Scale that consists of 14 items (eg, “I am regularly able to do what I do best”) [52,53]; and *symptoms* using the following interviewer-administered assessments: the Scale for the Assessment of Positive Symptoms that consists of 34 items in four symptom domains (hallucinations, delusions, bizarre behavior, and positive formal thought disorder) [54]; the Scale for the Assessment of Negative Symptoms that includes 25 items in five symptom domains (affective flattening or blunting, alogia, avolition-apathy, anhedonia-asociality, and attention) [55]; the Brief Psychiatric Rating Scale that includes 24 items assessing psychiatric symptoms such as somatic concern, anxiety, depression, and suicidality [46]; and the Calgary Depression Scale that includes nine items assessing depressive symptoms such as depressed mood, hopelessness, and suicide [56].

#### Qualitative Measures

Qualitative data on the acceptability, safety, and impact of the intervention was mainly obtained at the 4 weeks follow-up through the *HoryzonsCa Meet-up and Focus Group* discussion meeting.

The research assistant took field notes on any additional comments from participants related to their experiences and perspectives of the intervention during the *HoryzonsCa Initial Interview and Orientation Meeting* (which occur at baseline) and the HoryzonsCa Exit Interview (which occur at the 8 weeks follow-up). Moreover, participants were asked open-ended questions at the end of the HC-AUSI-Q, which were conducted during the *HoryzonsCa Exit Interview*.

At the *HoryzonsCa Initial Interview and Orientation Meeting* (60 minutes), participants completed the baseline measures (as described in previous sections), received an introduction to the website including review of its Terms of Use, and were provided with password information to log into the website using a pseudonym when logged in. They were invited to complete 3 to 5 activities (eg, identifying strengths and values through a card sorting activity and writing a short post in the café) and had the opportunity to ask questions and make comments about the intervention platform. The research assistant took field notes on participants’ initial impressions and questions about the website, concerns raised by the participant, and any challenges observed in navigating the intervention platform.

The *HoryzonsCa Meet-Up and Focus Group* (120 minutes) occurred midway through the 8 weeks of follow-up. We invited all participants to this Meet-up and Focus Group Meeting, and aimed to have approximately 4 to 8 participants each session. The focus groups were facilitated by the project lead with the support of a research assistant. Before having a focused discussion on the platform, the facilitator (who does not have any clinical role or therapeutic relationship with the participants) welcomed participants, provided an overview of the meet-up objectives, and provided logistic information (eg, duration of group and scheduled breaks). After that, participants were invited to share their experiences and perspectives of the system, and feedback on factors that support or hinder its use. Specifically, the topics of the focus group were general impressions of the HoryzonsCa intervention platform (eg, likes and dislikes), how easy it is to use, how useful the intervention platform has been for well-being, how the intervention platform can be improved to better meet the needs of Canadian youth, and any other suggestions in implementing and evaluating HoryzonsCa. The interview guides for the focus groups were adapted from our phase 1 adaptation study protocol, which was informed by the eHealth Adaptation Framework [31]. The facilitator encouraged an open discussion on the perceptions of the platform. A moderator was then invited to the room to present participants with the key components of the platform and provide tips on its use. At the end of the meeting, the lead facilitator reiterated the purpose of the focus group, summarized what was said, described the next steps, and gave participants the opportunity to bring up discussion points that were not addressed previously but which were of importance to them. These meetings were audio recorded.

During the *HoryzonsCa Exit Interview* (30 minutes), the TAUC-Q and the clinical measures used for the initial interview and the HC-AUSI-Q were completed. The research assistant took field notes including any additional comments that the participant provided related to the website or participation in the project.

#### Feasibility Measures

We also collected data on recruitment rates, appropriateness of eligibility criteria, and the project team’s experience of preparing for and implementing the intervention (based on team meeting notes) mainly to inform the feasibility and design for conducting a larger implementation study in the future.

### Data Management

This study is currently in the data management and analysis phase. The quantitative data from the outcome measures was entered into an Excel file (Microsoft Corporation). All outcome measures at baseline and at the 8 weeks follow-up are either self-reported or interviewer-administered, and those that were completed by the participant were checked by the interviewer for completeness, as sometimes questions are not answered simply because of rushing through, filling in errors, or oversight. Any missing data will be discussed using a team approach to first understand its nature (eg, participant nonresponse, research assistant error in data entry, or participant dropout). We will consider the extent and the type of the missing data (eg, missing completely at random, missing at random, and missing not at random), and then determine the best approach (eg, multiple imputation or regression imputation) to handle the missing data in consultation with a statistician [57]. Once all participants completed the *HoryzonsCa Exit Interview*, website use data was exported to an Excel file.

The qualitative data from the focus group audio recordings will be transcribed and anonymized. The field notes from the *HoryzonsCa Initial Interview and Orientation Meeting* and the *HoryzonsCa Exit Interview* taken by the research assistant will be typed by the research assistant. All qualitative data will be uploaded into the latest version of Atlas.ti software (ATLAS.ti Scientific Software Development GmbH), a coding software package.

### Analysis Plan

Following the convergent mixed methods model, the quantitative and qualitative data will first be analyzed separately and then considered for an integrated analysis of the findings [58]. The quantitative data (including website use data) will first be assessed using descriptive statistics (eg, frequencies). Specifically, to evaluate the acceptability and safety of HoryzonsCa, we will analyze quantitative feedback from the HC-AUSI-Q and website use by calculating proportions (ie, percentage of participants who indicate agree or strongly agree for specific items related to acceptability and safety; percentage of participants with at least 4 log-ins over the 8 weeks follow-up). To assess the potential efficacy of HoryzonsCa, paired samples *t* tests will be conducted on social functioning and clinical measures, and within-group effect sizes (Cohen *d*) will be reported for statistically significant changes between baseline and the 8 weeks follow-up. We will make Bonferroni corrections to reduce the inflation of alpha for multiple comparisons and determine a minimal clinically important difference to detect important changes over time. In addition, exploratory analysis of potential meditators and moderators of treatment effects will be conducted; for example, we will analyze the associations between website use and treatment effects to estimate the moderating role of website use. We will conduct the checks of assumptions prior to conducting analysis, including normality of the data, and will transform the data if needed. Two members of the research team (eg, a postdoctoral fellow and a research assistant) will review data quality (eg, missing data) and determine the best approach (eg, multiple imputation) to handle the missing data in consultation with a statistician and senior members of the research team. Quantitative data analysis will be supported using SPSS (IBM Corp; or R [R Foundation for Statistical Computing]).

Qualitative data (including qualitative feedback from the HC-AUSI-Q and the qualitative data from the interviews and focus groups) will be analyzed and reviewed for themes related to acceptability, perceived benefits, safety, barriers, and facilitators of using HoryzonsCa. Two members of the research team will review all the transcripts, codevelop a coding framework, and conduct a thematic analysis [59] in consultation with the project lead. These will be identified based on their salience with the research objectives and in relation to patterned responses. The coding categories will reflect questions asked during the interview and perspectives that emerged frequently in the data. Qualitative data analysis for the focus groups will be supported using the latest version of Atlas.ti software, a coding software package.

## Results

This study was funded by the Brain and Behavior Research Foundation (United States), Quebec Health Research Funding Agency (Canada), and the Canada Research Chairs Program (Canada), and was approved by the Research Ethics Board of the Centre intégré universitaire de santé et de services sociaux de l'Ouest-de-l'Île-de-Montréal on April 11, 2018 (#IUSMD 17-54). The study was registered as a clinical trial at [60] (ISRCTN43182105). Recruitment was initiated on May 10, 2018, and data collection occurred between August 16, 2018, to April 29, 2019. A total of 48 individuals were approached for the study, from which 20 were excluded (17 declined to participate, 1 was not reachable, 1 did not meet inclusion criteria, and 1 met exclusion criteria). The 28 remaining individuals provided informed consent and were invited to complete a baseline assessment, from which 4 did not attend for various reasons (eg, no longer interested, no longer attending clinic, or not feeling well). Among the 24 participants that completed the baseline assessment, 3 dropped out of the study before being given access to the intervention, and 1 was excluded from the intervention soon after team discussion of the baseline assessment due to not meeting inclusion criteria (ie, clinical stability). Upon completion of the baseline assessment and confirmation of eligibility criteria, a final sample of 20 participants were given access to the intervention and none of these were lost at 8 weeks follow-up. Participants were recruited over a 9-month duration (which was 3 times longer than our originally anticipated 3-month timeline), with a recruitment rate of approximately 3 participants per month. A total of 9 participants attended the focus group meetings of approximately 120 minutes each session (4 attended more than once). No adverse events related to the intervention occurred during the live pilot implementation. Further details on the results are expected to be submitted for publication in 2021.

## Discussion

### Importance of This Study

Psychotic disorders can have a profound impact on the individual, their caregivers, and society with regard to a loss of quality of life and productivity. Despite advances in early intervention, there remain ongoing challenges in treating this population, including preventing relapse and service disengagement, and sustaining improvements in symptoms and functioning over the long term. As such, accessible, sustainable, and engaging psychosocial services are needed to optimize care and outcomes for this population. The COVID-19 pandemic and public health guidelines for social distancing have compounded the urgent need for new models of service delivery to provide specialized services to patients with FEP. This study addresses this gap in health service research through leveraging HoryzonsCa, an online intervention with the potential to support recovery and prevent relapse in patients with FEP, during and post COVID-19.

In this paper, we have described the study protocol for pilot-testing HoryzonsCa, and it is the first study to implement and evaluate a live version of this online intervention in a Canadian context. This study is distinguished from previous research on Horyzons, as it is the first to be based on a systematic adaptation process [30,31] that considers geographical, cultural, and health care contexts. There is evidence to suggest that embarking on an adaptation process of an eHealth intervention (eg, considering language, culture, and context) can contribute positively to its adoption and effectiveness [31].

The Canadian context is distinguished from the Australian context in several ways that highlight the importance of Canadian-specific adaptation and testing of Horyzons. For example, there are differences in terms of history of colonization, such that Australia has a history of British colonization and Canada has both a British and French history, rendering certain health care settings particularly in Montreal to provide care in a bilingual context. This has implications for the adaptability of all features of the website intervention in terms of translation. There are also differences in the use of English terms and colloquialisms [31], and systems-level differences in community resources and how mental health services are organized and delivered. Indeed, based on our phase 1 research, we adapted several aspects of the intervention in relation to these differences, detailed in our previous publication, including but not exclusive to terms and colloquialisms, safety and moderation protocols, need help resources, terms of use, list of trigger words that will automatically be flagged by the system as indicating risk, and change to content and resources pertaining to employment/studying/volunteering to be in alignment with Canadian norms [30].

Furthermore, in comparison to Australia, the Canadian context lags in terms of implementation of digital health innovations [61]. Moreover, peer support legislation, policy, training, and provision is uneven across Canada [62]. These factors influenced our implementation of the intervention, particularly in relation to the hiring and training of the moderation team. For example, in previous Australian research on Horyzons, the clinician moderators were already working at the recruitment site delivering face-to-face services, with part of their time allocated to providing online moderation for Horyzons, whereas in the current context, the clinicians were ambivalent about making online moderation as part of their role, which may be due to the lack of experience and training in the delivering of online interventions [30]. As such, for this pilot study we decided to recruit a moderator external to the setting. Additionally, we had a limited pool of trained and experienced peer support worker workforce to recruit from and had turnover of 2 peer support workers (for reasons unrelated to the intervention) before being able to recruit a peer support worker that completed the duration of the project. However, it is noteworthy that this worker had limited experience in the delivery of online interventions and no employed experience as a peer support worker. As such, this pilot testing provides key information on the factors to consider in the implementation of this complex intervention (including recruitment and training of moderators and participant recruitment, retention, and engagement with the intervention). We will further detail and interpret these aspects of implementation using sociotechnical theory in our upcoming results report. In summary, through this study, we will gain insights into the acceptability, safety, and potential efficacy of HoryzonsCa, as well as key information that will support decision-making regarding scaling up the implementation and evaluation of this online intervention in Canada and abroad.

### Strengths and Limitations

There are several strengths to this research. First, in terms of preparing the intervention, we made adaptations to Horyzons to optimize its transferability from Australia to Canada, using a systematic approach and an eHealth adaptation framework, prior to its implementation [30,31]. The mixed methods design in this study is expected to provide an in-depth understanding of user experience and perspectives of HoryzonsCa. This mixed methods approach allows researchers to obtain a comprehensive view about the user experience and evaluate the potential impact of implementing such digital health innovations in Canadian health care settings.

Limitations of this study include a small sample size (N=20) and a single group, pre-post design. Due to this small sample size and a lack of a control group, we may not be able to detect treatment effects and impact of the intervention. Additionally, due to the short duration of follow-up (ie, 8 weeks), our findings regarding the acceptability, safety, and potential efficacy of HoryzonsCa will need to be interpreted with caution. Furthermore, our approach to exclude participants that score as moderately severe to extremely severe on suicidality (ie, due to recent suicidal ideations and behaviors, to mitigate the need for urgent intervention on a platform that is not monitored 24/7, and to reduce potential overexposure to comments that may be disruptive or distressing for other participants in the intervention) is not necessarily supported by predictive research and could even potentially exclude participants who may benefit from such an intervention [63,64]. Future research on HoryzonsCa should consider how such an online intervention platform can facilitate a recovery-oriented approach for all participants despite their score on a suicidality assessment while at the same time optimizing the safety of the online group environment. In addition, although the field of digital mental health is rapidly growing [23,61], we acknowledge that the limited evidence on the implementation of digital mental health interventions for people diagnosed with severe mental health disorders such as psychosis in Canada may limit the scope of our interpretations regarding the results. Nonetheless, the findings will help inform decision-making for scaling up the evaluation on HoryzonsCa to a larger implementation study.

### Conclusions

Considering daily use of ICTs among young people and an increasing need for new models of mental health service delivery in the context of the COVID-19 pandemic, research on digital health innovations such as online psychosocial interventions are needed to prevent relapses and support long-term recovery in young people experiencing FEP. However, limited attention has been given to how digital health innovations can be implemented and evaluated in Canada in the context of early interventions for psychosis. In this study, we have reported on the protocol for pilot-testing HoryzonsCa as a follow-up to our phase 1 adaptation study [30,31]. Specifically, this pilot study will provide preliminary evidence on the acceptability (including actual use), safety, and potential efficacy of HoryzonsCa for Canadian young adults with FEP, and an in-depth understanding of user experience and perspectives of HoryzonsCa (eg, perceived barriers and facilitators to using the platform). Our pilot study will inform the development of a research protocol (eg, including pre-post effect sizes on a number of variables conceptually targeted by the intervention, statistical power calculation, or sample size) for a larger implementation study of HoryzonsCa and that also considers sociotechnical factors pertaining to implementation as well as the experiences and perspectives of clinicians and peer support workers on moderating the intervention.

## Appendix

Multimedia Appendix 1 Safety protocol and withdrawal criteria.

Multimedia Appendix 2 Schedule of assessments.

## Abbreviations

FEP: first-episode psychosis
HC-AUSI-Q: Horyzons-Canada Acceptability, Usability, Safety, and Impact Questionnaire
HoryzonsCa: Horyzons-Canada
ICT: information and communication technology
IT: information technology
PEPP: Prevention and Early Intervention Program for Psychosis
TAM: Technology Acceptance Model
TAUC-Q: Technology Access, Use, and Competency Questionnaire
