# Supplementary material for: A Digital Health Innovation to Prevent Relapse and Support Recovery in Youth Receiving Specialized Services for First-Episode Psychosis: Protocol for a Pilot Pre-Post, Mixed Methods Study of Horyzons-Canada (Phase 2)
Source: JMIR Res Protoc. 2021 Dec 7;10(12):e28141. doi: 10.2196/28141 (PMC8693205; doi:10.2196/28141)
Supplement: Multimedia Appendix 1 [file resprot_v10i12e28141_app1.docx]

**Multimedia Appendix 1.** Safety protocol and withdrawal criteria.

In terms of **online and clinical safety**, all new posts on the website will be monitored daily by the clinical moderator. Moderation will be conducted by a minimum of 1 mental health clinician with at least 3 years of relevant clinical experience. Moderation will occur according to a prescribed schedule. The platform will be moderated twice daily for a minimum total duration of 60 minutes (30 minutes per each session), and brief checking will also occur on the weekend, twice daily for a total duration of 30 minutes (15 minutes each session).

**Clinical risk** will be managed through manual and automated procedures. Information related to clinical risk include posts made by participants which disclose evidence of psychotic, depression or suicidal symptoms; participants’ concerns, reports or complaints on posts made by other users; risk or self-harm related words automatically detected and blocked by the system; and presence of early warning signs of relapse detected by the regular monitoring of symptoms implemented within HoryzonsCa. Any detected increased risk will activate the HoryzonsCa pilot study safety protocol. System and privacy protection will be monitored by the study programmer. The moderator will have the administrative authority to respond to online reports (eg, remove offending material from the system) and automated emails. The moderators will be supported by a senior clinical-research team that will include the medical director/lead psychiatrist at the recruitment site.

In terms of **system security features**, the system incorporates visible emergency guidelines and contact information on every webpage; electronic reporting system (participants can click on a “report button” that sends automatic alerts to the Moderator’s interface regarding any issues they see with any of the posts online (eg, harmful posts and/or potential change in risk status requiring follow-up); automatic alerts can be programmed to be received as text messages directly to the clinician moderators; automatic word screening function which detects information consistent with increased risk of relapse or suicide or potentially harmful communication. The system has a list of key words (that can be customized) that it recognizes and responds to as follows: blocking messages that include pre-identified key words from appearing in the system, alerting the user with a message that if they are in need of urgent help, who to contact and also that the message can be rephrased if it was blocked by error, alerting the moderators that a message has been blocked and the moderators can decide either to block or unblock the message and contact the user if necessary. Potential problems associated with participating in the system in the event of deterioration in the mental state of a participant have also been considered and may result in the temporary suspension of individual accounts. Because participants may develop heightened concerns about communications that they have previously posted within the system once they have been enrolled, the system has been designed so that any participant can temporarily "hide" their profile and all of their on-line communications at any time.

**Textbox.** Withdrawal criteria.

| **Withdrawal from the trial will occur:**   - If participation in the study interferes with appropriate clinical management of risk, including risk of harm to self or others (as judged by the treating clinicians and/or senior researchers) - If serious adverse events that could be associated with the online intervention develop - If participants fail to comply with the terms of use of the online intervention - At the request of the participant or at the discretion of the lead investigators |
| --- |

Ratings for the withdrawal criteria will be made by the study RA in consultation with the lead investigators. Any signs of possible deterioration in mental state (eg, risk to self, deterioration in symptoms) that are observed by the clinician (or peer support) moderator will be discussed with the study RA and lead investigators, with appropriate follow up as per the safety protocol. The lead investigators will have the responsibility for deciding to withdraw participants from this pilot study, and this decision will be communicated to the participant by the treatment team.
