# Supplementary material for: A Digital Health Innovation to Prevent Relapse and Support Recovery in Youth Receiving Specialized Services for First-Episode Psychosis: Protocol for a Pilot Pre-Post, Mixed Methods Study of Horyzons-Canada (Phase 2)
Source: JMIR Res Protoc. 2021 Dec 7;10(12):e28141. doi: 10.2196/28141 (PMC8693205; doi:10.2196/28141)
Supplement: Multimedia Appendix 2 [file resprot_v10i12e28141_app2.docx]

**Multimedia Appendix 2.** Schedule of Assessments.

| **Assessment** | **Baseline** | **4 weeks** | **8 weeks** |
| --- | --- | --- | --- |
| HoryzonsCa Initial Interview and Orientation Meeting |  |  |  |
| Socio-demographic Questionnaire |  |  |  |
| Technology Access, Use, and Competency Questionnaire (TAUC-Q) |  |  |  |
| Social and Occupational Functioning Assessment Scale |  |  |  |
| Personal and Social Performance Scale |  |  |  |
| Clinical Global Impression Scale |  |  |  |
| Multidimensional Scale of Perceived Social Support |  |  |  |
| Self-Esteem Rating Scale |  |  |  |
| Strengths Knowledge Scale |  |  |  |
| Strengths Use Scale |  |  |  |
| Scale for the Assessment of Positive Symptoms |  |  |  |
| Scale for the Assessment of Negative symptoms |  |  |  |
| Brief Psychiatric Rating Scale |  |  |  |
| Calgary Depression Scale |  |  |  |
| HoryzonsCa Meet-up and Focus Group^a^ |  |  |  |
| HoryzonsCa Exit Interview (including the TAUC-Q^b^ and the clinical measures used for the HoryzonsCa Initial Interview, and the Horyzons-Canada Acceptability, Usability, Safety, and Impact Questionnaire) |  |  |  |

^a^All participants were invited to participate in the HoryzonsCa Meet-Up and Focus Group, among which 9 accepted the invitation and attended. A total of 4 focus groups were conducted.

^b^Technology Access, Use, and Competency Questionnaire.
